# Measurement of extracellular volume fraction using magnetic resonance-based conductivity tensor imaging

**DOI:** 10.3389/fphys.2023.1132911

**Published:** 2023-02-13

**Authors:** Bup Kyung Choi, Nitish Katoch, Ji Ae Park, Jin Woong Kim, Tong In Oh, Hyung Joong Kim, Eung Je Woo

**Affiliations:** ^1^ Department of Biomedical Engineering, Kyung Hee University, Seoul, Republic of Korea; ^2^ Division of Applied RI, Korea Institute of Radiological and Medical Science, Seoul, Republic of Korea; ^3^ Department of Radiology, Chosun University Hospital and Chosun University College of Medicine, Gwangju, Republic of Korea

**Keywords:** electrical conductivity, extracellular volume fraction, conductivity tensor imaging, giant vesicle, magnetic resonance imaging

## Abstract

Conductivity tensor imaging (CTI) using MRI is an advanced method that can non-invasively measure the electrical properties of living tissues. The contrast of CTI is based on underlying hypothesis about the proportionality between the mobility and diffusivity of ions and water molecules inside tissues. The experimental validation of CTI in both *in vitro* and *in vivo* settings is required as a reliable tool to assess tissue conditions. The changes in extracellular space can be indicators for disease progression, such as fibrosis, edema, and cell swelling. In this study, we conducted a phantom imaging experiment to test the feasibility of CTI for measuring the extracellular volume fraction in biological tissue. To mimic tissue conditions with different extracellular volume fractions, four chambers of giant vesicle suspension (GVS) with different vesicle densities were included in the phantom. The reconstructed CTI images of the phantom were compared with the separately-measured conductivity spectra of the four chambers using an impedance analyzer. Moreover, the values of the estimated extracellular volume fraction in each chamber were compared with those measured by a spectrophotometer. As the vesicle density increased, we found that the extracellular volume fraction, extracellular diffusion coefficient, and low-frequency conductivity decreased, while the intracellular diffusion coefficient slightly increased. On the other hand, the high-frequency conductivity could not clearly distinguish the four chambers. The extracellular volume fraction measured by the spectrophotometer and CTI method in each chamber were quite comparable, i.e., (1.00, 0.98 ± 0.01), (0.59, 0.63 ± 0.02), (0.40, 0.40 ± 0.05), and (0.16, 0.18 ± 0.02). The prominent factor influencing the low-frequency conductivity at different GVS densities was the extracellular volume fraction. Further studies are needed to validate the CTI method as a tool to measure the extracellular volume fractions in living tissues with different intracellular and extracellular compartments.

## 1 Introduction

Electrical conductivity is known to be affected by various tissue characteristics, such as cell density, cell size, extracellular matrix, membrane characteristics, water content, and the concentration and mobility of ions (Grimnes and Martinsen, 2015; Sajib et al., 2018; Katoch et al., 2019). The structural features of a biological tissue also affect its mobility (Choi et al., 2020). When cells are aligned toward a certain direction, the movements of ions in the extracellular fluid are more hindered along a direction perpendicular to the direction of cell alignments (Assaf and Basser, 2005; Katoch et al., 2019). In this situation, the tissue may exhibit anisotropic property (Tuch et al., 2001; Jeong et al., 2017; Chauhan et al., 2018). Since cell membranes only block low-frequency currents, some structural characteristics, such as cell swelling or changes in cellularity, can only be detected at low frequencies (Gabriel et al., 2009; Jahng et al., 2021). Since ions can move around cells at low frequency, their effective mobility is decreased, which in turn decreases the conductivity of that region (Gabriel et al., 1996; Gabriel et al., 2009; Choi et al., 2020). Therefore, low-frequency conductivity can provide information about changes in the extracellular space such as the extracellular volume fraction.

Homeostatic imbalance is one of the major causes of diseases (Sokolosky and Wargovich, 2012). Changes in extracellular volume could be an effective biomarker of a certain disease state such as fibrosis, edema, and cell swelling (Padhani et al., 2009). Cellular inflammation is a hallmark of many major diseases, such as neurological diseases and tumors (Chen, 2018). Tissues undergo microscopic structural changes as proliferating tumor cells take up more space (Jiang et al., 2017). As long as the cell membranes remain intact, the extracellular volume decreases, thus resulting in a decreased low-frequency conductivity value. However, when cells rupture, the extracellular volume increases, which leads to an increased low-frequency conductivity value.

Edema is a common response to various forms of injuries associated with extracellular space changes (Ho et al., 2012). In general, edema is an abnormal accumulation of extracellular water resulting from malfunction of the physiologic mechanisms that regulate total body water, circulating intravascular volume, and the maintenance of appropriate concentrations of cellular electrolytes (Oh and Baum, 2018). The conventional MR imaging methods, such as T1-weighted, T2-weighted, and diffusion-weighted imaging, focused on evaluating target tissues by comparing the MR contrasts of the tissues (Duong et al., 2001; Soria et al., 2020). However, until now, there have been few attempts to directly image and measure changes in extracellular space caused by cellularity due to the limitations of imaging methods. Measurements of extracellular volume changes inside tissues would provide valuable information for understanding the tissue conditions associated with disease progression.

Low-frequency conductivity tensor imaging (CTI) using an MRI has been developed to improve the limitations of existing MR-based conductivity imaging (Sajib et al., 2018; Wu et al., 2018; Katoch et al., 2019). Without injecting currents into the imaging object, CTI utilizes information on intracellular and extracellular compartments to produce both low-frequency and high-frequency conductivity images. After acquiring a high-frequency conductivity image using B1 mapping (Katscher et al., 2009; Voigt et al., 2011; Lee et al., 2016), a low-frequency conductivity tensor image is assembled by integrating multi-b-value diffusion imaging data from which intracellular and extracellular water diffusion coefficients, and extracellular volume fraction are estimated (Zhang et al., 2012). Several studies using a conductivity phantom have reported on the potential clinical utilities of CTI (Katoch et al., 2019; Choi et al., 2020; Jahng et al., 2021; Marino et al., 2021).

The extracellular volume fraction is one of the key parameters that need to be measured in CTI. In addition, it has its own clinical utilities if it can be measured reliably. The purpose of this study was to experimentally verify CTI parameters, with a specific focus on extracellular volume fraction, by using a conductivity phantom with position-dependent extracellular volume fractions. Such a phantom could be constructed using giant vesicle suspension (GVS) chambers with different vesicle densities. After describing how to construct such a phantom, we will analyze the reconstructed CTI parameters, such as high-frequency conductivity, extracellular volume fraction, extracellular diffusion coefficient, intracellular diffusion coefficient, and low-frequency conductivity of the GVS chambers. The reconstructed values of the low- and high-frequency conductivities of GVS chambers will be compared with the *in vitro* measurements of conductivity spectra using an impedance analyzer. The measured extracellular volume fraction using CTI will also be compared to those measured using a spectrophotometer.

## 2 Materials and methods

### 2.1 Giant vesicle suspension

**FIGURE 1:**
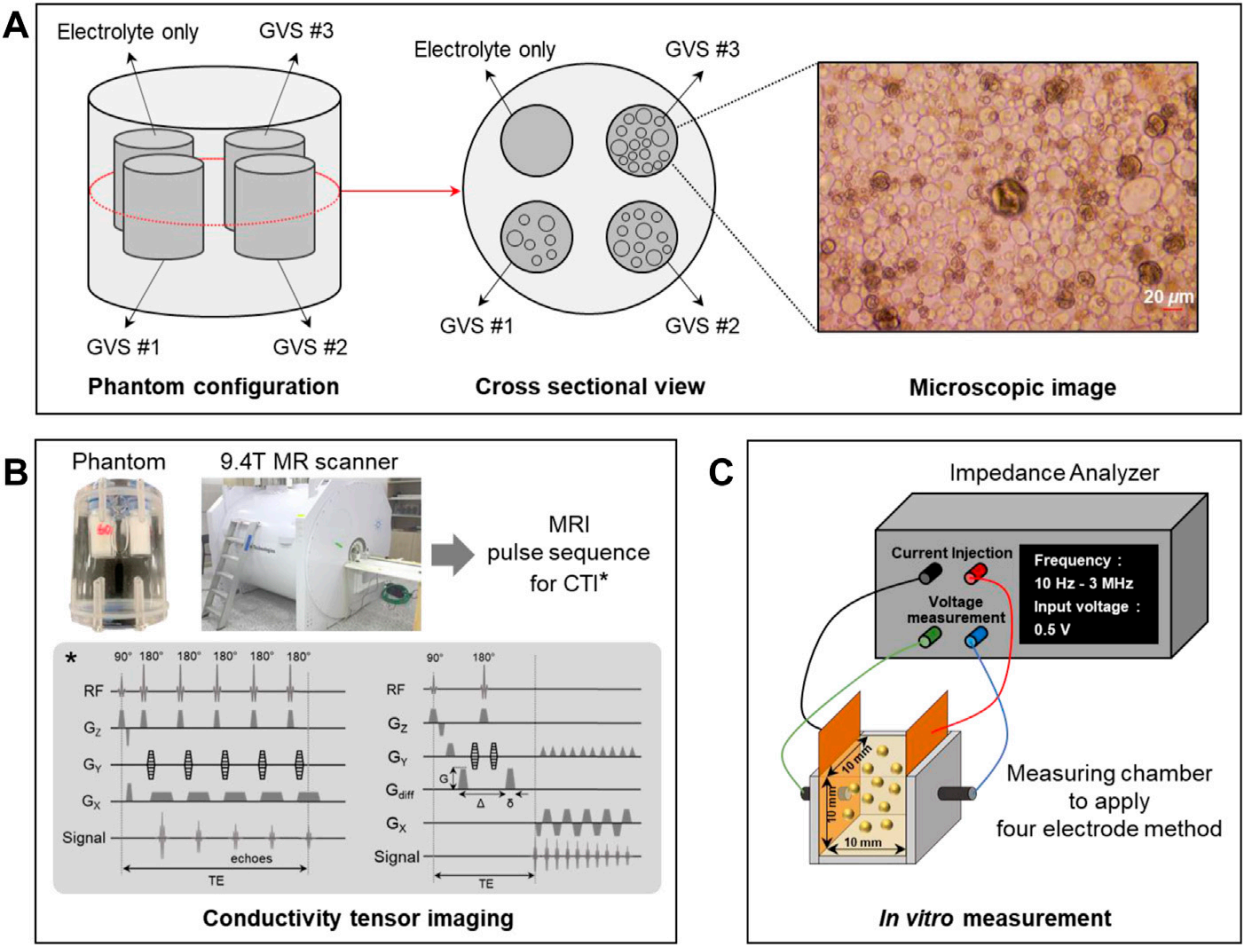
Phantom preparation **(A)**, experimental setup for CTI experiment using a 9.4T MRI scanner **(B)**, and *in vitro* measurement of conductivity spectra using an impedance analyzer **(C)**.

Giant vesicle suspension (GVS) was prepared using the reverse phase method (Moscho et al., 1996; Choi et al., 2020). A phospholipid (Avanti Polar Lipids, United States) was dissolved in chloroform (Sigma-Aldrich, United States) to make a lipid solution at 30 mg/ml under an argon atmosphere. This lipid solution was positioned at the bottom of a 1 L round flask, which then had 400 *µ*l of methanol added to it. Next, 0.9% NaCl electrolyte was carefully added to the lipid solution to form a two-phase. The flask was installed in a rotary evaporator (N-1300V-W, EYELA, Japan) to maintain two-phase prior to rotation. The installed flask was then heated in a bath to remove chloroform at 47°C under vacuum. When the chloroform began to evaporate, the flask was rotated at 10 rpm for 15 min and 60 rpm for 10 min. The evaporated chloroform was captured with a nitrogen trap to avoid explosion and poisoning. During the evaporation of chloroform, the phospholipids were assembled to form a lipid membrane as giant vesicles. The giant vesicle solution was centrifuged at 1500 rpm for 10 min to divide the giant vesicle and electrolyte. After removing electrolytes from the solution, the final result of suspension had a volume fraction of about 90% by visual observation of their microscopic images. The size of the giant vesicles was about 20 *μ*m on visual inspection from the microscopic image (Figure 1A).

### 2.2 Phantom preparation

Acrylic phantom with a 40 mm diameter and a 60 mm height was used for the CTI imaging experiment. The changes in extracellular volume were controlled by the GVS chambers with the following four different densities: electrolyte only and low (GVS #1), middle (GVS #2), and high (GVS #3) densities of GVS (Figure 1A). The chamber of electrolyte only was NaCl solution of 8 g/L whereas the other GVS chambers were composed of the electrolyte with three different densities of GVS. The designed extracellular volume fractions of electrolyte only, GVS #1, GVS #2, and GVS #3 were 1, 0.6, 0.4, and 0.2, respectively. The chambers containing the four densities of GVS were 9 mm in diameter each. The outsides of the chambers were filled with fomblin oil (Y/L 25/6, Solvay, Italy) to prevent susceptibility artifacts during imaging experiments.

### 2.3 Conductivity tensor imaging experiment

The CTI experiment was performed using a 9.4T MR scanner (Agilent Technologies, United States) with a single-channel body coil (Figure 1B). After positioning the phantom inside the bore of the MR scanner, a multi-echo multi-slice (MEMS) spin-echo MR pulse sequence was applied to acquire B1 phase maps to reconstruct high-frequency conductivity (*σ*_*H*_) images of the phantom. The imaging parameters were as follows: repetition time (TR)/echo time (TE) = 1500/15 ms, number of echoes = 6, number of averaging = 5, slice thickness = 0.5 mm, number of slices = 5, matrix size = 128 × 128, field-of-view (FOV) = 60 × 60 mm^2^, and scan time = 16 min. The single-shot spin-echo echo-planar (SS-SE-EPI) imaging sequence was used for multi-b-value diffusion-weighted imag (DWI). The imaging parameters were as follows: TR/TE = 1800/39 ms, number of b-values = 13 (0, 50, 150, 300, 500, 700, 1000, 1400, 1800, 2200, 2600, 3000, 3500), number of averaging = 1, slice thickness = 0.5 mm, number of slices = 5, matrix size = 128 × 128, FOV = 60 × 60 mm^2^, and scan time = 96 min and 36 s.

### 2.4 Conductivity tensor image reconstruction

Conductivity tensor images were reconstructed using an MRCI toolbox which is available at http://iirc.khu.ac.kr/toolbox.html (Sajib et al., 2017). The raw data was extracted from the k-space of the MR spectrometer. To minimize geometrical mismatches, the B1 phase maps and DWI were registered with the anatomical T2-weighted images after denoising and bias corrections. The B1 phase map, which is the spatial sensitivity distribution of the applied RF coil measured *via* MRI, is used to obtain *σ*_*H*_ (Katscher et al., 2009). From the MEMS data obtained after imaging experiment, the multiple echoes were combined to achieve a higher signal-to-noise ratio (SNR) using a weighting factor. The optimized phase maps were used to reconstruct *σ*_*H*_ (Gurler and Ider, 2017). The multi-b-value DWI data were corrected for eddy-current effects and geometrical distortions. The averaged images at b = 0 were linearly co-registered to the magnitude images of MEMS data, and the affine transformation matrix was used to non-linearly co-register the multi-b-value DWI (Smith, 2002). The conductivity of extracellular space can be defined as the product of ion concentration and mobility of charged particles. The following CTI formula was used for all conductivity tensor image reconstructions (Sajib et al., 2018):

$$\sigma_{L}=\alpha \sigma_{e}=\alpha \bar{c_{e}}\mu_{e}$$ (1)

where *σ*_*L*_ is the low-frequency conductivity; *α* is the extracellular volume fraction; *σ*_*e*_ is the conductivity of extracellular space; $\bar{c_{e}}$ is the ion concentration; $\mu_{e}$ is the ion mobility. The apparent extracellular ion concentration $\bar{c_{e}}$ can be estimated as suggested by Sajib et al, (2018).

$$\bar{c_{e}}=\frac{\sigma_{H}}{\alpha d_{e}^{w}+\left(1-\alpha \right)d_{i}^{w}\beta }$$ (2)

where *β* is the ion concentration ratio of the intracellular and extracellular spaces; *d*_*e*_^*w*^ and *d*_*i*_^*w*^ are the extracellular and intracellular water diffusion coefficients, respectively. Since the ion concentration inside and outside of the giant vesicles are almost similar (*β* = 1), the low-frequency isotropic conductivity *σ*_*L*_ can be expressed as follows:

$$\sigma_{L}=\frac{\alpha \sigma_{H}}{\alpha d_{e}^{w}+\left(1-\alpha \right)d_{i}^{w}\beta }d_{e}^{w}$$ (3)

The details of the conductivity tensor reconstruction procedures followed those outlined in the works of Katoch et al, (2019).

### 2.5 Conductivity spectra measurement using impedance analyzer

To validate *σ*_*L*_ and *σ*_*H*_ in the CTI results, the conductivity spectra of the four densities of GVS were directly measured using an impedance analyzer (SI1260A, METEK, United Kingdom) (Figure 1C). To this end, 1 ml of each solution extracted from the different densities of GVS was placed in a 10 × 10 × 10 mm^3^ chamber equipped with the electrodes, and the conductivity spectra were measured using a four-electrode method. The frequency range of the conductivity spectra was from 10 Hz to 3 MHz.

### 2.6 Volume fraction measurement using spectrophotometer

**FIGURE 2:**
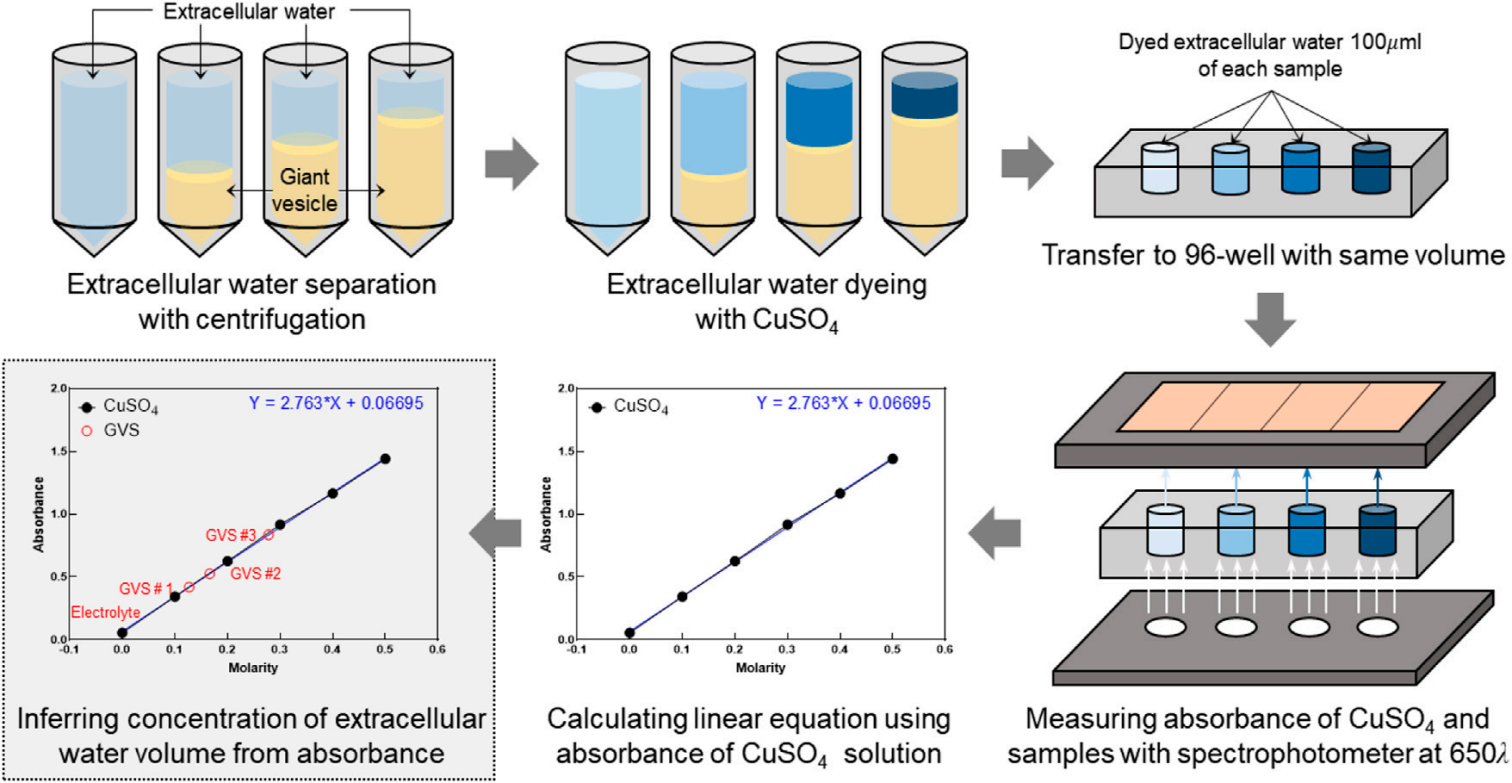
Experimental procedure to measure extracellular volume fraction using a spectrophotometer. Extracellular volume fractions of samples with four densities of GVS were experimentally calculated from the absorbance and concentration of the colored solutions.

For comparison with the results of CTI parameters, *α* was experimentally calculated from the absorbance and concentration of the colored solutions using a spectrophotometer (Absorbance 96, Byonoy GmbH, Germany) (Figure 2). Six solutions of CuSO_4_ with different molarity values (0–0.5 M) were prepared to calculate the linear equation of absorbance at 650 nm and concentration. A linear fitting was applied using the acquired absorbance as shown in Figure 2. To calculate *α* of four GVS densities, we put 200 *µ*l of 0.5M CuSO_4_ solution into each of the 1 ml samples of the four GVS densities. Here, four densities of giant vesicles were visually confirmed by microscopic images after being put into the suspension. After vortexing those samples, the extracellular water in the four samples was separated by centrifugation. The absorbance was measured in the extracted extracellular water of samples, and the concentrations of CuSO_4_ were calculated from those measured absorbances. Then, the amount of extracellular water in each sample was calculated from the decreased concentrations of CuSO_4_. Finally, *α* of electrolyte only and different densities of GVS were estimated from the extracellular water volume of each sample.

## 3 Results

### 3.1 CTI of four different densities of GVS

**FIGURE 3:**
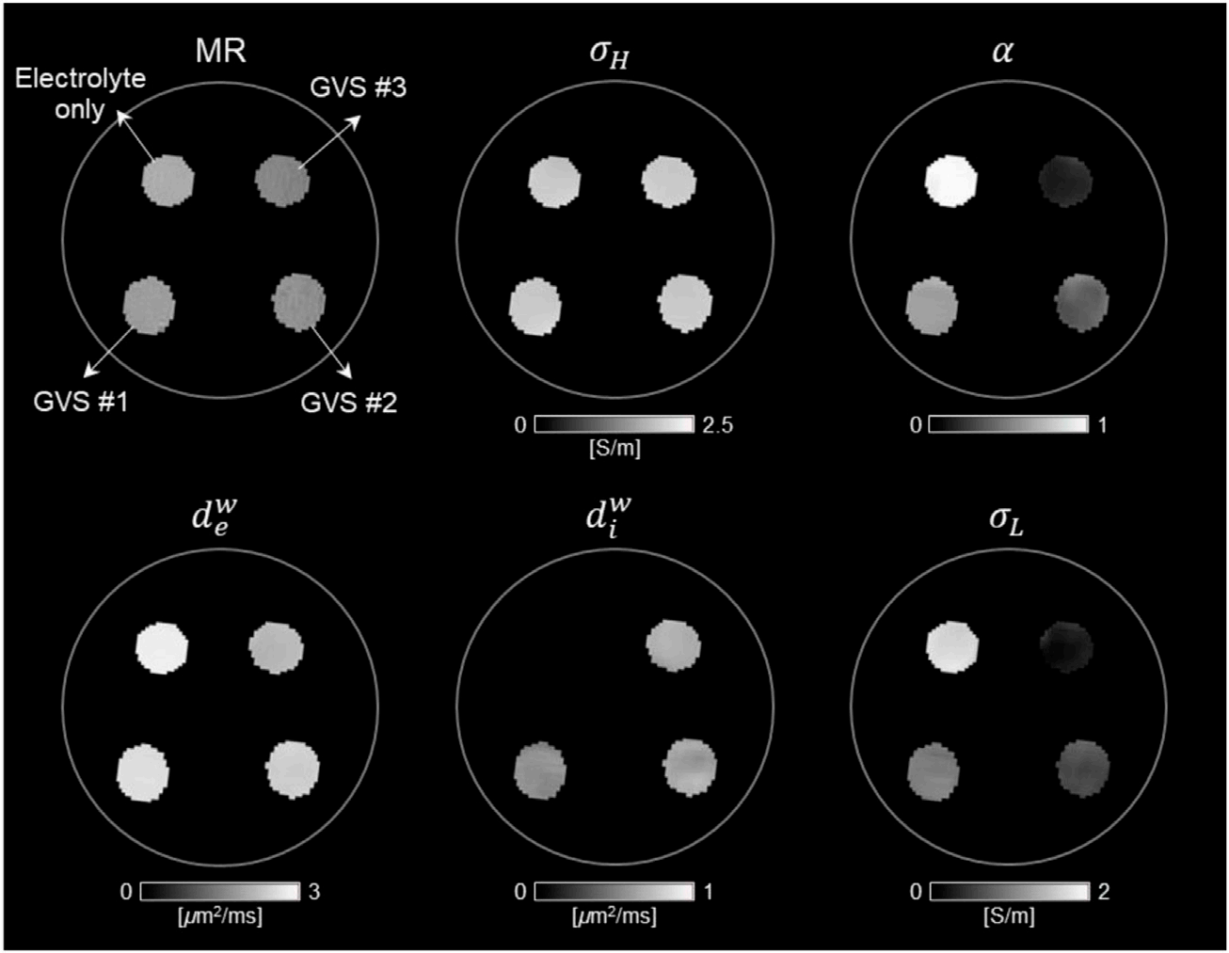
Conductivity tensor images of phantom with GVS chambers using a 9.4T MRI. The contrast changes in CTI parameters, such as high-frequency conductivity (*σ*_*H*_), extracellular volume fraction (*α*), extracellular diffusion coefficient (*d*_*e*_^*w*^), intracellular diffusion coefficient (*d*_*i*_^*w*^), and low-frequency conductivity (*σ*_*L*_), were attributed to the density of GVS.

Figure 3 shows the reconstructed CTI parameters obtained from the imaging experiment of the GVS phantom. The T2-weighted MR image shows the morphology of the GVS phantom. The signal intensity was found to be highest in the chamber with electrolyte only, but there was no clear difference in signal intensity between the chambers with different GVS densities. The *σ*_*H*_ image did not show a clear difference in terms of contrast between the four different densities of GVS. However, *α*, *d*_*e*_^*w*^, and *σ*_*L*_ all showed clear differences in contrast depending on the densities of GVS. Specifically, the contrast of these parameters was decreased as the density of GVS increased. Meanwhile, the contrast of *d*_*i*_^*w*^ was slightly increased with increasing of GVS density. There was no contrast in the chamber of electrolyte only due to the absence of GVS.

### 3.2 Estimation of extracellular volume changes by CTI parameters

**FIGURE 4:**
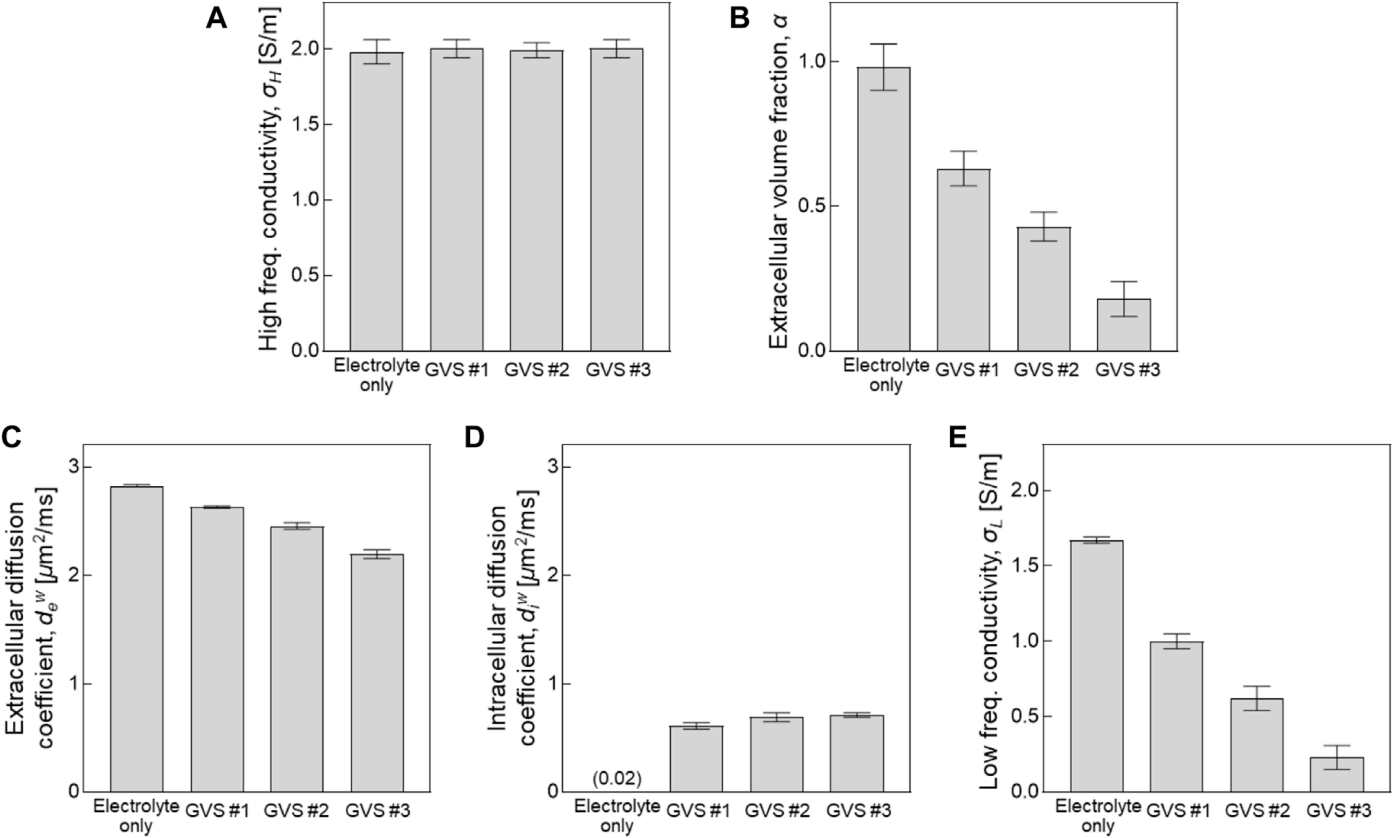
Comparison of CTI parameters depending on different densities of GVS. Bar graph represent the values of each parameter obtained from the entire area of the four chambers.

Figure 4 and Table 1 present comparisons of the CTI parameters according to the four different densities of GVS. The bar graphs present the measured values of each of the CTI parameters from the entire area of the four chambers. There were no differences in *σ*_*H*_ between the four different densities of GVS. A significant decrease with the increase in GVS density was measured in *α*, *d*_*e*_^*w*^ and *σ*_*L*_. In contrast *d*_*i*_^*w*^ was slightly increased with increasing of GVS density. The value of the electrolyte only was 0.02 [*μ*m^2^/ms]. When reconstructing CTI parameters, the factor that most affected to *σ*_*L*_ in this experiment was *α*. The designed extracellular volume fractions of electrolyte only, GVS #1, GVS #2, and GVS #3 were 1, 0.6, 0.4, and 0.2, respectively. The corresponding extracellular volume fractions in the CTI parameter were measured at 0.98 ± 0.01, 0.63 ± 0.02, 0.43 ± 0.05, and 0.18 ± 0.02, respectively.

**TABLE 1:** Summary of CTI parameters with different GVS densities. The relative error of conductivity was calculated between the low-frequency conductivity of CTI and the *in vitro* measurement at 10 Hz.

	CTI parameters					*In vitro* measurement	Relative error [%]
	$\sigma_{H}$ [S/m]	α	$\begin{array}{l} d_{e}^{w}\\ \left[\mu m^{2}/\text{ms}\right] \end{array}$	$\begin{array}{l} d_{i}^{w}\\ \left[\mu m^{2}/\text{ms}\right] \end{array}$	$\sigma_{L}$ [S/m]	$\sigma_{L}$ [S/m] at 10Hz	
Electrolyte	1.98 ± 0.08	0.98 ± 0.01	2.82 ± 0.01	0.02 ± 0.01	1.67 ± 0.02	1.62 ± 0.00	3.23
GVS #1	2.00 ± 0.06	0.63 ± 0.02	2.63 ± 0.03	0.61 ± 0.03	1.00 ± 0.05	1.06 ± 0.00	5.32
GVS #2	1.99 ± 0.05	0.40 ± 0.05	2.39 ± 0.07	0.69 ± 0.04	0.62 ± 0.08	0.59 ± 0.00	4.51
GVS #3	2.00 ± 0.06	0.18 ± 0.02	2.17 ± 0.03	0.71 ± 0.02	0.23 ± 0.08	0.23 ± 0.00	0.28

$*\;Relative\;error\;\left(\%\right)=\left|\frac{\sigma_{L}\left(in\;vitro\right)-\sigma_{L}\left(CTI\right)}{\sigma_{L}\left(in\;vitro\right)}\right|\times 100$

### 3.3 Validation of measured conductivity and extracellular volume fraction

**FIGURE 5:**
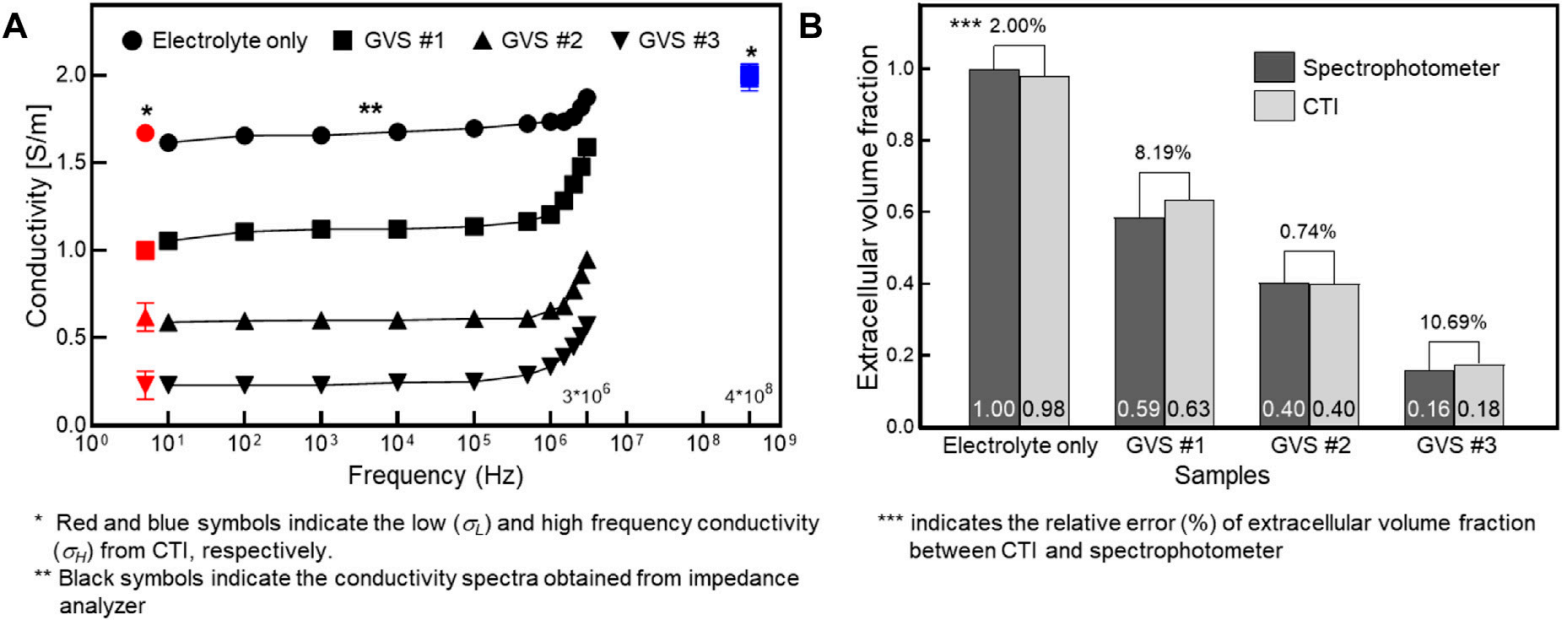
Comparison of the conductivity between CTI and *in vitro* measurement **(A)** and comparison of the extracellular volume fraction between CTI and spectrophotometer **(B)** depending on four different GVS densities.

Figure 5 shows a comparison of the conductivity between CTI and *in vitro* measurement and a comparison of *α* between CTI and the spectrophotometer result. In Figure 5A, *σ*_*L*_ obtained from CTI showed that the conductivity was highest in the electrolyte only and decreased with increasing density of GVS. In the conductivity spectra obtained from an impedance analyzer of less than 1 MHz, the measured conductivity was also decreased with increasing GVS density. The measured values above 1 MHz showed the same pattern, but the absolute conductivity values were increased due to the frequency-dependency. In contrast, *σ*_*H*_ (400 MHz at 9.4T MRI) obtained from CTI showed almost similar values due to the absence of the cell membrane effect at high-frequency. The low-frequency conductivities of the four different densities in CTI were 1.67, 1.00, 0.62, and 0.23 S/m, respectively, and in the *in vitro* measurement at 10 Hz, they were 1.62, 1.06, 0.59, and 0.23 S/m, respectively. The relative error between the CTI and *in vitro* measurements at 10 Hz were 3.23, 5.32, 4.51, and 0.28%, respectively (Table 1).

The extracellular volume fractions obtained from the CTI and spectrophotometer are compared in Figure 5B. Table 2 summarizes the extracellular volume fractions calculated from the results of the spectrophotometer. The CuSO_4_ concentrations of the four samples were found to be 0, 0.127, 0.166, and 0.278 mM, respectively, by substituting the absorbance of the GVS into the linear equation. The volume of extracellular water was estimated from the concentrations of CuSO_4_, and the values were 1000.0, 585.9, 402.6, and 159.3 ml in each sample. From the estimated volumes of extracellular water, the extracellular volume fractions of the samples with electrolyte only, GVS #1, GVS #2, and GVS #3 were measured at 1.000, 0.586, 0.403, and 0.159, respectively. The extracellular volume fraction was decreased as the density of GVS increased in both methods. The relative error values between the two methods were 2.00, 8.19, 0.74, and 10.69%, respectively (Figure 5B).

**TABLE 2:** Summary of measuring extracellular volume fraction according to different densities of GVS obtained from spectrophotometer.

	Spectrophotometer				CTI
	Absorbance (at 650λ)	Molarity of CuSO_4_ (mM)	*ECW (ml)	**EVF	EVF
Electrolyte	0.068	0.000	1000.0	1.000	0.980
GVS #1	0.419	0.127	585.9	0.586	0.634
GVS #2	0.526	0.166	402.6	0.403	0.401
GVS #3	0.836	0.278	159.3	0.159	0.176

∗Indicates the volume of extracellular water, ∗∗is extracellular volume fraction.

## 4 Discussion

Changes in cellularity affect the intracellular and extracellular spaces within tissues (Huang et al., 2017; Jiang et al., 2017). As a disease progresses, changes in cellularity and extracellular space could occur in a complex manner. Both individual factors and their relations should be analyzed to interpret such changes in tissue condition. However, it is often quite difficult to distinguish such individual factors in the intracellular and extracellular spaces by comparing conventional MR images and histopathologic findings (Padhani et al., 2009). DWI has been suggested as a method with which to measure the cellularity through the assessment of apparent diffusion coefficient (ADC) values in tumors (Le Bihan et al., 2006; Aso et al., 2009; Harkins et al., 2009; Surov et al., 2017; Roberts et al., 2020). Tumor cellularity and the shape of the extracellular space both affect water diffusion (Chen et al., 2013). The diffusivity of water molecules is restricted in microenvironments with high cellularity because high cellularity reduces the ratio of the extracellular to intracellular space in a given area of tissues (Huang et al., 2017). The ADC values were used to distinguish the diffusion properties of intracellular and extracellular compartments. The multi-b-values and multi-diffusion gradient directions have been widely studied to detect diffusion coefficients and the intra- and extra-weighted volume fractions (Le Bihan et al., 2006; Zhang et al., 2012). A number of theories have been proposed to account for the changes in water diffusion in the intracellular and extracellular spaces (Harkins et al., 2009; Novikov et al., 2019).

Several studies have reported the potential of CTI, which can accurately provide information about the microstructure of tissue due to their contrast mechanism, which primarily originates from the concentration and mobility of ions (Katoch et al., 2019; Choi et al., 2020; Lee et al., 2020; Jahng et al., 2021; Marino et al., 2021). When acquiring CTI data, *σ*_*H*_ image includes information on the ion concentration inside tissues, and multi-b-value DWI includes information on the intracellular and extracellular compartments. In this study, CTI method was applied and verified as a reliable tool to measure the changes in the intracellular and extracellular spaces depending on the change in density from the cell-mimicking model of giant vesicle suspension. From the results of a phantom CTI experiment, CTI parameters such as *σ*_*H*_, *α*, *d*_*e*_^*w*^, *d*_*i*_^*w*^, and *σ*_*L*_ were reconstructed to distinguish contrast changes with different GVS densities.

The results of *σ*_*H*_ provided mixed information about the extracellular and intracellular compartments because there was no cell membrane effect. Since the inside and outside of the giant vesicle membrane were designed to have the same conductivity, *σ*_*H*_ did not show contrast changes depending on different GVS densities in our results. When controlling the changes in extracellular volume depending on different GVS densities, the designed extracellular volume fraction of chambers with electrolyte only, GVS #1, GVS #2, and GVS #3 were 1, 0.6, 0.4, and 0.2, respectively. From *α* in CTI parameters, the chamber with electrolyte only was close to one because there was no intracellular space. The other chambers were measured at 0.63, 0.40, and 0.18, respectively. *α* showed a linear decrease with increasing GVS density. Compared to the spectrophotometry results, the relative error of *α* ranged from a minimum of 0.74% to a maximum of 10.69%. This can be estimated to be an experimental error from the volume change of GVS due to the diffusion of water molecules, which is caused by the difference in ion concentration between CuSO_4_ solution and GVS during spectrophotometry measurement. Although the maximum error was 10.69%, the difference in *α* between the two methods ranged from a minimum of 0.02 to a maximum of 0.04.

When reconstructing CTI parameters, the most influential factor in *σ*_*L*_ was *α*. Since the ion concentration of the intracellular and extracellular spaces was similar in the giant vesicles (*β* = 1), the prominent factor influencing *σ*_*L*_ was *α* in the CTI formula. In our imaging results, *σ*_*L*_ linearly decreased as the density of GVS increased, which was the same pattern seen in the result of *α*. The changes in *σ*_*L*_ in the CTI results were consistent with *in vitro* measurements using an impedance analyzer. Since *σ*_*L*_ provides mostly ionic information about the extracellular compartment (Lee et al., 2020), the decrease in *α* with increasing GVS density can be considered an affecting factor. The *d*_*e*_^*w*^ and *d*_*i*_^*w*^ showed conflicting results depending on the GVS density. Similar to the results of *σ*_*L*_ and *α*, *d*_*e*_^*w*^ decreased as the density of GVS increased. It can be inferred that the diffusion of water molecules in the extracellular space is hindered as the GVS density increases. In contrast, *d*_*i*_^*w*^ was slightly increased with increasing GVS density. Theoretically, *d*_*i*_^*w*^ should not be changed as the cell density increases or decreases because the only factor that could change the value of *d*_*i*_^*w*^ in this experiment was the cell size. It can therefore be assumed that this may be caused by the non-uniformity of the giant vesicle size or by merging each other within the GVS.

## 5 Conclusion

In this study, the changes in the extracellular volume depending on the different densities of the cell-mimicking model were imaged using the CTI method. The advantage of the CTI method is that it simultaneously provides information on both the intracellular and extracellular spaces and the cellularity. Using a cell-mimicking model of GVS, the changes in extracellular volume fraction depending on different GVS densities were analyzed in terms of the CTI parameters. The extracellular volume fraction was a key parameter in measuring CTI because the electrolytes inside and outside of the giant vesicle have similar ionic concentration. As the vesicle density increased, the extracellular volume decreases, thus resulting in a decreased low-frequency conductivity value. The low-frequency conductivity and extracellular volume fraction of the CTI parameters were matched with those measured by the impedance analyzer and spectrophotometer. Further animal and clinical studies are needed to evaluate the clinical utility of CTI in assessing intracellular and extracellular volumes of live cells with different intracellular and extracellular compartments.

## Data Availability

The original contributions presented in the study are included in the article/supplementary material, further inquiries can be directed to the corresponding authors.

## Abbreviations

CTI, Conductivity tensor imaging; GVS, Giant vesicle suspension; *σ*_*L*_, Low-frequency conductivity; *σ*_*H*_, High-frequency conductivity; *α*, Extracellular volume fraction; *d*_*e*_^*w*^, Extracellular diffusion coefficient; *d*_*i*_^*w*^, Intracellular diffusion coefficient; *β*, Ion concentration ratio of intracellular and extracellular spaces; *σ*_*e*_, Conductivity of extracellular space; $\bar{c_{e}}$ , Ion concentration; $\mu_{e}$ , Ion mobility.
